# Noncompliance With Safety Guidelines as a Free-Riding Strategy: An Evolutionary Game-Theoretic Approach to Cooperation During the COVID-19 Pandemic

**DOI:** 10.3389/fpsyg.2021.646892

**Published:** 2021-03-16

**Authors:** Jose C. Yong, Bryan K. C. Choy

**Affiliations:** ^1^Nanyang Business School, Nanyang Technological University, Singapore, Singapore; ^2^School of Social Sciences, Singapore Management University, Singapore, Singapore

**Keywords:** evolutionary game theory, decision-making, COVID-19, free riding, evolutionary psychology, cooperation, public goods, public goods dilemma

## Abstract

Evolutionary game theory and public goods games offer an important framework to understand cooperation during pandemics. From this perspective, the COVID-19 situation can be conceptualized as a dilemma where people who neglect safety precautions act as free riders, because they get to enjoy the benefits of decreased health risk from others’ compliance with policies despite not contributing to or even undermining public safety themselves. At the same time, humans appear to carry a suite of evolved psychological mechanisms aimed at curbing free riding in order to ensure the continued provision of public goods, which can be leveraged to develop more effective measures to promote compliance with regulations. We also highlight factors beyond free riding that reduce compliance rates, such as the emergence of conspiratorial thinking, which seriously undermine the effectiveness of measures to suppress free riding. Together, the current paper outlines the social dynamics that occur in public goods dilemmas involving the spread of infectious disease, highlights the utility and limits of evolutionary game-theoretic approaches for COVID-19 management, and suggests novel directions based on emerging challenges to cooperation.

## Introduction

The numerous articles on COVID-19 to date represent an impressive effort to come to terms with, inform about, and manage the crisis. We suggest that evolutionary game theory (EGT) and, more specifically, the public goods perspective can significantly add to this discussion and enhance our understanding of human behavior during a global pandemic. From this perspective, people who disregard safety procedures, such as wearing masks and maintaining social distance, can be understood as free riders because they get to enjoy the benefits of communal safety despite not doing their part to uphold it (Cato et al., 2020). Indeed, there have been calls for more studies on free riding to make sense of uncooperative behavior during COVID-19 (Naso, 2020, p. 72), not least because “continued noncompliance eventually will degrade any benefit associated with these [safety] practices.” Such behaviors also do injustice “especially to high-risk groups, people with diseases, and the health workforce trying to treat these patient groups and save their lives” (Paakkari and Okan, 2020, p. e249). As such, the current paper aims to introduce the broader ideas of EGT, describe free-riding behavior in the context of COVID-19, and outline possible mechanisms that may inhibit them. We also consider factors beyond free riding that reduce compliance rates, such as socioeconomic inequality and the emerging problem of conspiracy theory-driven noncooperation that appears immune to free-rider suppression mechanisms. In so doing, we broaden the accessibility of EGT to a wider audience, highlight the utility and limits of EGT for COVID-19 research, and encourage more work in this important area.

## Evolutionary Game Theory and Pandemics

Game theory provides a framework to understand strategic decision-making under interdependent payoff structures known as “games” in which a player’s outcomes depend on the decisions of other players. The “public goods game” is one such well-studied game-theoretic scenario that affords an examination of people’s motivations to partake in the maintenance of public goods, or commodities and services that are available to all members of a society (Olson, 1965; Hardin, 1968). The optimal solution occurs when everyone equally contributes to the provisioning of public goods. However, there are two ways that free riding – or the exploitation of others’ cooperation to avoid cooperating – may arise (Apesteguia and Maier-Rigaud, 2006). When the maintenance of a public good (e.g., a clean shared bathroom) depends on the voluntary contributions of a critical number of individuals, an incentive exists for people to avoid bearing those costs when there are sufficient people already contributing, because those who do not contribute are seldom excluded from the benefits. Another form of free riding can occur when public goods are “common pool resources” with a carrying capacity (e.g., a fruit garden). As consumption of resources beyond the carrying capacity limit can lead to rapid depletion of the stock, people are incentivized to take more than a fair share. Yet, if too many people engage in such free-riding behaviors, the sustainability of the public good will be undermined to the detriment of all. Such situations characterize the public goods dilemma, where individuals choose between cooperating to maintain the public good at some personal cost versus free riding if there are already enough cooperative group members. Game-theoretic models indicate that even if individuals are initially cooperative, the inability to exclude free riders renders cooperation an unviable long-term strategy (Perc et al., 2017). Experiments have further confirmed that a lack of measures to suppress free riding pushes individuals toward noncooperation over time (e.g., Fehr and Gächter, 2000; Herrmann et al., 2008; Ibuka et al., 2014).

EGT applies game-theoretic models to biological populations to determine the strategies that evolved for organisms to maximize payoffs in fitness terms (i.e., survival and reproduction; Maynard Smith and Price, 1973; Tooby et al., 2006). Through modeling public goods games, two key adaptive strategies have been identified. On the one hand, humans may have evolved a proclivity to free ride due to the high fitness payoff of selfishness when public goods are available and costs on noncooperative behavior are low. On the other hand, as there are considerable benefits associated with public goods being available, humans also may have evolved psychological adaptations that are geared toward suppressing free riding to facilitate the continued provision of such goods (Tooby et al., 2006). These dynamics can be extended to not only explain difficulties behind real-world scenarios, including voting, law enforcement, and environmental behavior, but also derive solutions that take into account our evolved human nature (e.g., Fehr and Gächter, 2000; Tooby et al., 2006; Bowles and Gintis, 2011; Li et al., 2020).

EGT has also been used to examine the social tensions that occur when a public goods dilemma arises in circumstances involving infectious disease. According to the theory, “herd immunity” emerges when a large enough number of people get vaccinated, which effectively slows and eventually stops the spread of infections (cf. “vaccination game,” Fu et al., 2011).^1^ As herd immunity becomes a public good, the following social dilemma arises: people can choose between getting vaccinated and contributing to herd immunity versus not getting vaccinated and still benefitting from herd immunity. Because cooperation contributes to the collective benefit of the group at some individual cost (e.g., the hassle or expense of getting vaccinated and potential side effects), whereas a free-riding strategy maximizes individual benefits at no cost (i.e., escaping infection without vaccinating oneself), an incentive to avoid vaccination exists. Experiments indeed show that as a population’s vaccination rate increased, people increasingly decided not to vaccinate themselves (Ibuka et al., 2014). The vaccination game also reveals asymmetries in free-riding incentives and risks at different levels of analysis. For instance, the incentive to free ride varies as a function of age as older people are more susceptible to disease compared to younger individuals (Piraveenan et al., 2020). Despite vaccinations being free, quick, and well tolerated by recipients in developed countries, the urge to free ride can strongly emerge in such populations as they tend to have lower viral prevalence and higher rates of vaccination compared to less developed countries (Sharma et al., 2019).^2^ At the same time, our highly globalized world entails that the vulnerability of richer nations hinges critically on the ability of poorer nations with less developed immunization programs to achieve herd immunity (Bollyky and Bown, 2021).^3^

While free riding can emerge as a dominant strategy in the vaccination game, it can also be mitigated in various ways. For example, the isolation of infectious persons and the quarantine of exposed persons can be instrumental in managing the spread of disease, especially when these measures are used jointly (Alam et al., 2020). Another suggestion is to lower the costs or increase the payoffs of cooperation through subsidies and incentive-based vaccination programs (Vardavas et al., 2007), though this approach appears to be effective only or especially under certain conditions, such as when group-based rather than individual-based incentives are used (Chapman et al., 2012), subsidies are targeted at appropriate subpopulations (Ding et al., 2018), vaccine efficacy is sufficiently high (Arefin et al., 2019), and/or information about the status of an infectious disease is publicly available (Ruan et al., 2012). Characteristics of social networks (e.g., heterogeneity) in the population (Kuga and Tanimoto, 2018), which play an important role in influencing the success of cooperative strategies, can also be exploited to enhance the effectiveness of measures and interventions.

## The Public Goods Dilemma in the Context of COVID-19

The COVID-19 situation can be similarly conceptualized as a public goods game, because a virus-free environment constitutes a public good whose upkeep depends on people’s joint commitment toward safety behaviors (Paakkari and Okan, 2020). Yet, because no one can be excluded from the benefits of a virus-free environment, the risk (and incentive) that people would choose not to cooperate and enjoy these benefits at the expense of others arises and culminates in the free-rider problem. Unwillingness to comply with public health guidelines has indeed emerged as a recurrent problem that severely undermines efforts to curtail the virus (Naso, 2020). For example, a substantial proportion of the American population refuses to wear masks (Kramer, 2020) despite evidence of their effectiveness in reducing infectiousness (Haischer et al., 2020). Community surveys also found that 39% of Americans do not intend to be vaccinated, with almost half of these individuals convinced that more information would not sway their decision (Funk and Tyson, 2020). These cases of noncompliance are not limited to the United States and they also include refusing to engage in social distancing (Murphy et al., 2020), resuming pre-pandemic social activities (Simonov et al., 2020), and failing to step up hygiene behaviors (Pan et al., 2020). As there is a level of social interaction that can be tolerated before the virus spreads exponentially in the population, people also have an incentive to engage in more social interaction than the carrying capacity allows, especially in the face of pandemic fatigue (Miller, 2020). The heterogenous effects of COVID-19 further contribute to differences in people’s readiness to observe safety. Younger individuals who expect to have less serious symptoms from infection, for instance, have weaker incentives to act prudently (Coroiu et al., 2020). Anonymity in a large group coupled with other citizens following safety recommendations also reduces individual accountability and creates a safe zone for free riding to occur (Paakkari and Okan, 2020). Beyond individual-level noncompliance, the inability of some countries to manage COVID-19 domestically because of lax policies and inadequate responses has also contributed to global ineffectiveness in disease management when travel bans are lifted due to pressure to resume flights (Hymas, 2020). We are only as strong as our weakest links – “even if the share of such people is small, the collective consequences can be dire. In sum, even if some people follow social distancing measures for self-preservation reasons alone, the social average is likely to be substantially lower than the level required to eradicate the pandemic” (Cato et al., 2020, p. 51).

## Recommendations to Reduce Free Riding and Increase Cooperation

EGT offers important insights on how the COVID-19 situation as a public goods dilemma could be tackled. Free riding has been shown to elicit negative emotions and perceptions of unfairness among cooperators, and humans seem naturally inclined toward identifying, punishing, and suppressing free-riding behaviors through social norms, sanctions, and imposing reputational costs, thus altering decision payoffs in favor of cooperation in public goods situations (Fehr and Gächter, 2000; Ostrom, 2000; Bowles and Gintis, 2011). For instance, vaccinated individuals showed less generosity toward their non-vaccinated counterparts who were perceived as violating the social contract, regardless of whether they came from the same or different social groups (Korn et al., 2020). As public goods confer significant fitness advantages, humans evolved a psychology that seeks to increase both the costs of free riding and the benefits of cooperation so that public goods can be sustained (Tooby et al., 2006; Cosmides et al., 2010). Knowledge of this evolved psychology can be useful in guiding the development of measures to protect a crucial public good like a virus-free and safe environment.

Using a game-theoretic approach to analyze COVID-19 behaviors, Cato et al. (2020) conceptualized safety benefits from social distancing (e.g., not dining out) as a public good and identified several social and psychological mechanisms that may inhibit free riding and increase people’s likelihood of maintaining social distance. Based on a survey of 2,177 Japanese participants, the authors found that those who agreed that “it is important to always avoid doing anything people would say is wrong” were more apt to limit unnecessary social activity, thus indicating that public shame can promote safe behavior particularly when relational harmony and social obligations are regarded as important. Participants who indicated that they valued helping others and making others happy were also likelier to observe safety regulations, suggesting that altruistic and prosocial orientations promote cooperativeness (cf. Böhm et al., 2016).

As not everyone is altruistic or norm-abiding, voluntary-based policies can lead to insufficient compliance (Korn et al., 2020), thus necessitating the use of sanctions and punishments (e.g., legal enforcement and penalties for violations) to impose costs on noncompliance (Cato et al., 2020). State-enforced punishments are also important when social norms disincentivize people from calling out free riders at a communal level, such as when individuals face retaliation when they urge others to observe safety (Raihani and Bshary, 2015; Porterfield, 2020). Indeed, pool punishment (e.g., third-party punishers such as the police) is often preferable over peer punishment (e.g., laypersons who sanction others’ misbehavior) as it facilitates the maintenance of order without disrupting the social fabric (Traulsen et al., 2012). That said, people tend to feel especially enraged when high-profile individuals such as politicians and celebrities get caught flouting rules and regulations (Bentham, 2020; Kok, 2020). As such individuals may exploit their influential status to escape punishment, anger can be adaptive in motivating laypersons to ensure that higher-status individuals remain accountable.

There are notable limits to the effectiveness of these mechanisms, in particular, the extrinsically motivated measures of shaming and punishment (Bénabou and Tirole, 2006; Jacquet et al., 2011). For example, worse outcomes may occur if shaming by ordinary citizens leads to stigmatization of infected persons, which can inadvertently make people hide their illness and hinder timely detection of the virus. In addition, the motivation to shame others can exacerbate intergroup tensions, such as when citizens utilize the COVID-19 situation to justify marginalizing outgroups (Habersaat et al., 2020; Prichard and Christman, 2020). Government-led punishment and enforcement can also be very costly. Punishment has proven to be a controversial strategy in the political context of a pandemic (Naso, 2020), especially when psychological reactance is triggered in individuals who misperceive the risks or have other priorities (May, 2005; Habersaat et al., 2020; we explore this issue further in the Discussion section). Moreover, the mobilization of police forces for surveillance and enforcement is a heavy expense when resources are needed across many other essential domains, and enforced lockdowns have also resulted in severe economic losses as the epicenters of infection are primarily metropolitan and commercial areas (Coibion et al., 2020). Considering these limitations, Cato et al. (2020) recommended the use of (1) voluntary- or nudge-based approaches as they carry low economic costs while preserving civil liberties and (2) moderate legal sanctions to address more serious cases of noncompliant behavior, but with some caveats: preferably when altruistic or other-regarding concerns are a feature of prevailing norms. As simple penalties such as fines can result in individuals feeling morally licensed to commit violations insofar as they pay the fee (Gneezy and Rustichini, 2000; Bowles and Polanía-Reyes, 2012), it is important that citizens care enough about how they are judged for reneging on social obligations for such punishments to have a desired effect.

## Other Game-Theoretic Considerations

Inefficacies in COVID-19 management can be additionally explored with game-theoretic scenarios beyond the public goods game. In particular, some researchers have focused on the hesitancy of governments in enacting difficult but necessary virus containment measures (e.g., stay-at-home orders and lockdowns), as well as noncooperation for reasons other than free riding. For instance, Kabir and Tanimoto (2020) argued that because strict stay-at-home measures can greatly impact people’s livelihoods, the cost of staying home (coupled with lockdown fatigue) can end up outweighing the risk of infection from going out. As individual-level decisions have a direct impact on the society-level effectiveness of stay-at-home orders, governments may refrain from implementing them because of anticipated low rates of compliance, especially from socioeconomically disadvantaged individuals who do not have the luxury of staying home (Blundell et al., 2020). Some governments may have also been hopeful that herd immunity from recoveries and vaccinations would allow them to avoid imposing such unpopular measures altogether (Weitz et al., 2020).

With rising numbers of cases and stretched health facilities, as well as the lack of a vaccine throughout 2020 and difficulties associated with achieving herd immunity for COVID-19 (Iwasaki, 2021; Sridhar and Gurdasani, 2021), government inaction became increasingly unviable. Hence, to increase people’s adherence to strict regulations, Kabir and Tanimoto suggested using social programs such as emergency-relief funds and unemployment insurance to lower the costs of compliance, particularly for lower-paid workers (cf. Wilson, 2020; Wong and Wong, 2021). As vaccines became available at the end of 2020, Piraveenan et al. (2020) argued that programs driving vaccination uptake will surpass other aspects like vaccine efficacy and isolation procedures in importance. Using EGT, social network analysis, and agent-based modeling, the authors proposed that individuals’ vaccination decision-making will be influenced by “demographics, physical location, the level of interaction, the health of the vaccine, epidemic parameters, and perceptions about the vaccine being introduced. Similarly, the decision-making of the government will be influenced by epidemic parameters, the nature of the vaccine being introduced, logistics, the management of human resources needed for the vaccination effort, and the amount of vaccine doses available” (Piraveenan et al., 2020, p. 11). In sum, holistic COVID-19 management would involve an appreciation of the many factors that calibrate payoffs so that both individual and governmental decisions shift toward safety.

## Discussion

A burgeoning literature has emerged in the wake of COVID-19 in recognition of the various lessons that can be gleaned from this experience, including studies of countries that have been relatively successful in managing the virus (e.g., Cousins, 2020; Wilson, 2020; Haslam et al., 2021). We suggest that more studies that view health safety as a public goods dilemma will prove highly illuminating and significantly contribute to disease management efforts. From this perspective, refusal to adhere to safety regulations reflects a free-riding strategy as noncooperative individuals capitalize on others’ willingness to incur the costs of practicing safe behaviors without bearing those costs themselves. Our review highlights a comprehensive set of recommendations derived from EGT. Apart from containment measures, such as mask wearing, social distancing, and quarantine requirements and, eventually when vaccines are available, targeted programs to drive vaccination uptake, societal conditions that can reduce free riding and increase compliance are also crucial. From the top down, the enforcement of penalties that are commensurate with the degree of violation with an eye on potential side effects is needed. From the bottom up, social norms that foster altruistic attitudes as well as concern for relational harmony and the fulfillment of social obligations should be cultivated as they can promote cooperation in a less heavy-handed manner while also enhancing the effectiveness of top-down measures. Finally, the payoffs of compliance can be increased through subsidies for the disadvantaged alongside various other economic incentives. These measures are compatible with our evolved psychology for cooperation and holding noncooperators accountable, especially when people feel vested in the protection of cherished public goods.

Some debates exist over the importance of punishment in free-rider prevention. The backlash against sanctions in some countries notwithstanding, some researchers have also suggested that cooperation can occur without the need for punishment, particularly when group returns from individual contributions are non-linear (e.g., Kameda et al., 2011; Yong et al., 2021). The COVID-19 situation, however, demonstrates some necessity for heavy sanctions, particularly when social norms promoting voluntary cooperation are lacking. Noncompliance appears more severe in countries with highly individualistic orientations and low obedience to authority – where outright defiance against mask wearing and other health safety directives is not uncommon – compared to countries with high levels of collectivistic orientation and authority deference (Tan and Li, 2020; Gelfand et al., 2021). As it is easier to mobilize collective effort when people care about the public good and are willing to work together under the instruction of the authorities, norms that promote concern for relational harmony and social obligations coupled with willingness to abide by government regulations may be most effective in eliciting compliance. That said, noncompliance also occurs in authoritarian, collectivistic countries, albeit more furtively through secret activities such as illegal gatherings (Lim, 2020). Such transgressions continue to underscore the importance of sanctions and punishment, as voluntary goodwill may be insufficient to sustain the cooperation that is crucial for safety and yet highly fragile at a collective level (Bowles and Gintis, 2011; Naso, 2020).

## The Emerging Role of Conspiratorial Thinking in Noncooperative Behavior

The growing problem of conspiracy theory-driven noncooperation is worth noting in the present context (Arnot et al., 2020; Prichard and Christman, 2020). As an epiphenomenon of adaptive rationalization mechanisms, conspiratorial thinking may be triggered as people struggle to gain a sense of control during crises such as COVID-19 (Yong et al., 2020). Distrust in societal institutions can further drive conspiratorial thinking and galvanize resistance against perceived threats (van Prooijen and van Vugt, 2018). Conspiracy theorists may, for instance, genuinely believe that COVID-19 is a hoax perpetuated by the “deep state,” leading them to view their noncompliance as heroic, those who comply as weak or ignorant, and their accusers as evil (Collins, 2020). As such noncooperators have a substantially distinct understanding of the public goods at stake, analyses of their actions from a social dilemma perspective may be inadequate or even inappropriate, rendering many of the aforementioned strategies to induce cooperation (e.g., social disapproval and punishments) ineffective. Indeed, punishments imposed on them may be interpreted as persecution for their beliefs, which can inadvertently strengthen their worldviews and drive the growth of fringe clusters (Arnot et al., 2020). Dealing with such noncooperators requires a consideration of the characteristics of social networks (Kuga and Tanimoto, 2018) as well as measures that are presently outside the scope of EGT, such as controlling the spread of misinformation (Yong et al., 2020), influencing normative behavior through group identification and peer intervention (Arnot et al., 2020), and reducing perceptions of environmental hostility and inequality (van Prooijen and van Vugt, 2018). As the difficulty of spurring cooperation from conspiracy theorists also shows, a limitation of the public goods perspective is that solutions based on free-rider suppression may be more applicable in some environments than in others. Thus, further research is needed to develop improved EGT models that can address the unprecedented challenges associated with modern developments in social dynamics, such as globalization and information technology (Li et al., 2020).

## Further Directions and Conclusions

In sum, we highlight both the relevance and limits of EGT for infectious disease situations and suggest that more studies, in particular, cross-national empirical investigations and research that extends EGT to novel scenarios, can contribute important insights on cooperation during COVID-19 and beyond. Further discussions on the complexities of applying EGT to design effective interventions in the COVID-19 context are also warranted as these are complex, iterative games that are concurrently happening in different ways around the world. Other evolutionarily driven directions may also enlarge our understanding of behaviors in pandemic events alongside EGT, including cooperation versus competition as a function of threat (van Prooijen and van Vugt, 2018), inclusive fitness (Arnot et al., 2020), and adaptive leadership styles (Luoto and Varella, 2021), as well as risky behavior as a function of dominance signaling (Ronay and von Hippel, 2010) or life history strategies (Corpuz et al., 2020). Some of the research in these areas has indeed refined what we know about behaviors during COVID-19. For instance, although younger individuals have generally been observed to be less cautious and less compliant than older individuals (Coroiu et al., 2020), research that carefully examined the effects of life history while controlling for age has shown that life history strategy, but not age, was associated with COVID-19 precautions and projected behaviors (Corpuz et al., 2020). Through an integration of these perspectives with EGT, we are more likely to achieve a holistic understanding of dynamic, interdependent behaviors during pandemics and encourage compliance with public health guidelines in ways that work with rather than go against our evolved human nature.

## Author Contributions

All authors listed have made a substantial, direct, and intellectual contribution to the work and approved it for publication.

### Conflict of Interest

The authors declare that the research was conducted in the absence of any commercial or financial relationships that could be construed as a potential conflict of interest.

## Footnotes

^1^It is important to note that herd immunity may be elusive or even impossible to achieve with COVID-19 because of the peculiar nature of the SARS-CoV-2 virus, including its various mutations and the propensity for reinfection (Iwasaki, 2021; Sridhar and Gurdasani, 2021; van Oosterhout et al., 2021). Hence, while this section discusses pandemic situations in general, unique features of the SARS-CoV-2 virus may render some of the traditional insights of EGT less applicable to the COVID-19 pandemic.

^2^Another way by which COVID-19 differs from previous pandemics is that while developed countries have historically had lower viral prevalence during pandemics, this is not the case for COVID-19 as global human development indices have been shown to be positively correlated with COVID-19 deaths (*N* = 168, *r* = 0.51; Luoto and Varella, 2021).

^3^Once more, this section serves to demonstrate how EGT has previously been used to analyze pandemic situations in general. Given the elusiveness of herd immunity for COVID-19, it is important to recognize limits to the applicability of the vaccination game for COVID-19.

## References

[ref1] Alam M. Kabir K. M. A. Tanimoto J. (2020). Based on mathematical epidemiology and evolutionary game theory, which is more effective: quarantine or isolation policy? J. Stat. Mech.: Theory Exp. 2020:033502. 10.1088/1742-5468/ab75ea

[ref2] Apesteguia J. Maier-Rigaud F. P. (2006). The role of rivalry: public goods versus common-pool resources. J. Confl. Resolut. 50, 646–663. 10.1177/0022002706290433

[ref3] Arefin M. R. Masaki T. Kabir K. M. A. Tanimoto J. (2019). Interplay between cost and effectiveness in influenza vaccine uptake: a vaccination game approach. Proc. Math. Phys. Eng. Sci. 475:20190608. 10.1098/rspa.2019.0608, PMID: 31892839 PMC6936611

[ref4] Arnot M. Brandl E. Campbell O. L. K. Chen Y. Du J. Dyble M.. (2020). How evolutionary behavioural sciences can help us understand behaviour in a pandemic. Evol. Med. Public Health 2020, 264–278. 10.1093/emph/eoaa038, PMID: 33318799 PMC7665496

[ref6] Bénabou R. Tirole J. (2006). Incentives and prosocial behavior. Am. Econ. Rev. 96, 1562–1678. 10.1257/aer.96.5.1652

[ref7] Bentham M. (2020). Metropolitan police warn celebrities over coronavirus rule flouting after Rita Ora party: police say ‘those with higher public profiles have even greater responsibility’ to stick to the rules. *Evening Standard*. Available at: https://www.standard.co.uk/news/crime/police-warning-celebrities-flout-covid-rules-rita-ora-b139226.html (Accessed December 28, 2020).

[ref8] Blundell R. Costa Dias M. Joyce R. Xu X. (2020). COVID-19 and inequalities. Fisc. Stud. 41, 291–319. 10.1111/1475-5890.12232, PMID: 32836542 PMC7362053

[ref9] Böhm R. Betsch C. Korn L. (2016). Selfish-rational non-vaccination: experimental evidence from an interactive vaccination game. J. Econ. Behav. Organ. 131, 183–195. 10.1016/j.jebo.2015.11.008

[ref10] Bollyky T. J. Bown C. P. (2021). The tragedy of vaccine nationalism. *Foreign Affairs*. Available at: https://www.foreignaffairs.com/articles/united-states/2020-07-27/vaccine-nationalism-pandemic (Accessed February 9, 2021).

[ref11] Bowles S. Gintis H. (2011). A cooperative species: Human reciprocity and its evolution. Princeton, NJ: Princeton University Press.

[ref12] Bowles S. Polanía-Reyes S. (2012). Economic incentives and social preferences: substitutes or complements? J. Econ. Lit. 50, 368–425. 10.1257/jel.50.2.368

[ref13] Cato S. Iida T. Ishida K. Ito A. McElwain K. M. Shoji M. (2020). Social distancing as a public good under the COVID-19 pandemic. Public Health 188, 51–53. 10.1016/j.puhe.2020.08.005, PMID: 33120232 PMC7425541

[ref14] Chapman G. B. Li M. Vietri J. Ibuka Y. Thomas D. Yoon H.. (2012). Using game theory to examine incentives in influenza vaccination behavior. Psychol. Sci. 23, 1008–1015. 10.1177/0956797612437606, PMID: 22810166

[ref15] Coibion O. Gorodnichenko Y. Weber M. (2020). The cost of the COVID-19 crisis: lockdowns, macroeconomic expectations, and consumer spending. NBER. 10.3386/w27141

[ref16] Collins B. (2020). How QAnon rode the pandemic to new heights—and fueled the viral anti-mask phenomenon. *NBC News*. Available at: https://www.nbcnews.com/tech/tech-news/how-qanon-rode-pandemic-new-heights-fueled-viral-anti-mask-n1236695 (Accessed February 5, 2021).

[ref17] Coroiu A. Moran C. Campbell T. Geller A. C. (2020). Barriers and facilitators of adherence to social distancing recommendations during COVID-19 among a large international sample of adults. PLoS One 15:e0239795. 10.1371/journal.pone.0239795, PMID: 33027281 PMC7540845

[ref18] Corpuz R. D’Alessandro S. Adeyemo J. Jankowski N. Kandalaft K. (2020). Life history orientation predicts COVID-19 precautions and projected behaviors. Front. Psychol. 11:1857. 10.3389/fpsyg.2020.01857, PMID: 32793087 PMC7393224

[ref19] Cosmides L. Barrett H. C. Tooby J. (2010). Adaptive specializations, social exchange, and the evolution of human intelligence. Proc. Natl. Acad. Sci. U.S.A 107, 9007–9014. 10.1073/pnas.0914623107, PMID: 20445099 PMC3024027

[ref20] Cousins S. (2020). New Zealand eliminates COVID-19. Lancet 395:1474. 10.1016/S0140-6736(20)31097-7, PMID: 32386582 PMC7252131

[ref64] Ding H. Xu J.-H. Wang Z. Ren Y.-Z. Cui G.-H. (2018). Subsidy strategy based on history information can stimulate voluntary vaccination behaviors on seasonal diseases J. Theor. Biol. 503, 390–399. 10.1016/j.physa.2018.03.003, PMID: 31678271

[ref21] Fehr E. Gächter S. (2000). Cooperation and punishment in public goods experiments. Am. Econ. Rev. 90, 980–994. 10.1257/aer.90.4.980

[ref22] Fu F. Rosenbloom D. I. Wang L. Nowak M. A. (2011). Imitation dynamics of vaccination behaviour on social networks. Proc. R. Soc. B Biol. Sci. 278, 42–49. 10.1098/rspb.2010.1107, PMID: 20667876 PMC2992723

[ref24] Funk C. Tyson A. (2020). Intent to get a COVID-19 vaccine rises to 60% as confidence in research and development process increases. *Pew Research Center*. Available at: https://www.pewresearch.org/science/2020/12/03/intent-to-get-a-covid-19-vaccine-rises-to-60-as-confidence-in-research-and-development-process-increases/ (Accessed February 19, 2021).

[ref25] Gelfand M. J. Jackson J. C. Pan X. Nau D. Pieper D. Denison E.. (2021). The relationship between cultural tightness-looseness and COVID-19 cases and deaths: a global analysis. Lancet Planet. Health. 10.1016/S2542-5196(20)30301-6, PMID: [Epub ahead of print] 33524310 PMC7946418

[ref26] Gneezy U. Rustichini A. (2000). A fine is a price. J. Leg. Stud. 29, 1–17. 10.1086/468061

[ref27] Habersaat K. B. Betsch C. Danchin M. Sunstein C. R. Böhm R. Falk A.. (2020). Ten considerations for effectively managing the COVID-19 transition. Nat. Hum. Behav. 4, 677–687. 10.1038/s41562-020-0906-x, PMID: 32581299

[ref28] Haischer M. H. Beilfuss R. Hart M. R. Opielinski L. Wrucke D. Zirgaitis G.. (2020). Who is wearing a mask? Gender-, age-, and location-related differences during the COVID-19 pandemic. PLoS One 15:e0240785. 10.1371/journal.pone.0240785, PMID: 33057375 PMC7561164

[ref29] Hardin G. (1968). The tragedy of the commons. Science 162, 1243–1248. 10.1126/science.162.3859.1243, PMID: 5699198

[ref30] Haslam S. A. Steffens N. K. Reicher S. Bentley S. (2021). Identity leadership in a crisis: a 5R framework for learning from responses to COVID-19. Soc. Issues Policy Rev. 15, 35–83. 10.1111/sipr.12075 PMC8013601 33821168

[ref31] Herrmann B. Thoni C. Gachter S. (2008). Antisocial punishment across societies. Science 319, 1362–1367. 10.1126/science.1153808, PMID: 18323447

[ref32] Hymas C. (2020). Airlines schedule major increase in flights in July as pressure mounts on ministers to ease quarantine. *The Telegraph*. Available at: https://www.telegraph.co.uk/politics/2020/05/30/airlines-schedule-major-increase-flights-july-pressure-mounts/ (Accessed December 28, 2020).

[ref33] Ibuka Y. Li M. Vietri J. Chapman G. B. Galvani A. P. (2014). Free-riding behavior in vaccination decisions: an experimental study. PLoS One 9:e87164. 10.1371/journal.pone.0087164, PMID: 24475246 PMC3901764

[ref34] Iwasaki A. (2021). What reinfections mean for COVID-19. Lancet Infect. Dis. 21, 3–5. 10.1016/S1473-3099(20)30783-0, PMID: 33058796 PMC7550040

[ref35] Jacquet J. Hauert C. Traulsen A. Milinski M. (2011). Shame and honour drive cooperation. Biol. Lett. 7, 899–901. 10.1098/rsbl.2011.0367, PMID: 21632623 PMC3210662

[ref36] Kabir K. M. A. Tanimoto J. (2020). Evolutionary game theory modelling to represent the behavioural dynamics of economic shutdowns and shield immunity in the COVID-19 pandemic. R. Soc. Open Sci. 7:201095. 10.1098/rsos.201095, PMID: 33047059 PMC7540740

[ref37] Kameda T. Tsukasaki T. Hastie R. Berg N. (2011). Democracy under uncertainty: the wisdom of crowds and the free-rider problem in group decision making. Psychol. Rev. 118, 76–96. 10.1037/a0020699, PMID: 20822292

[ref38] Kok Y. (2020). Ah boys to men actor Maxi Lim’s wedding faces probe for Covid-19 breaches. *The Straits Times*. Available at: https://www.straitstimes.com/singapore/ah-boys-actors-wedding-faces-probe-for-covid-19-breaches (Accessed December 28, 2020).

[ref39] Korn L. Böhm R. Meier N. W. Betsch C. (2020). Vaccination as a social contract. Proc. Natl. Acad. Sci. 117, 14890–14899. 10.1073/pnas.1919666117, PMID: 32541033 PMC7334515

[ref40] Kramer S. (2020). More Americans say they are regularly wearing masks in stores and other businesses. *Pew Research Center*. Available at: https://www.pewresearch.org/fact-tank/2020/08/27/more-americans-say-they-are-regularly-wearing-masks-in-stores-and-other-businesses/ (Accessed February 19, 2021).

[ref41] Kuga K. Tanimoto J. (2018). Impact of imperfect vaccination and defense against contagion on vaccination behavior in complex networks. J. Stat. Mech.: Theory Exp. 2018:113402. 10.1088/1742-5468/aae84f

[ref42] Li N. P. Yong J. C. van Vugt M. (2020). Evolutionary psychology's next challenge: solving modern problems using a mismatch perspective. Evol. Behav. Sci. 14, 362–367. 10.1037/ebs0000207

[ref43] Lim J. (2020). 12 foreigners deported and barred from Singapore for non-compliance with safe distancing measures. *The Straits Times*. Available at: https://www.straitstimes.com/singapore/courts-crime/12-foreigners-deported-and-barred-from-singapore-for-non-compliance-with-safe (Accessed December 28, 2020).

[ref44] Luoto S. Varella M. A. C. (2021). Pandemic leadership: sex differences and their evolutionary–developmental origins. Front. Psychol. 10.3389/fpsyg.2021.633862 PMC8015803 33815218

[ref45] May T. (2005). Public communication, risk perception, and the viability of preventive vaccination against communicable diseases. Bioethics 19, 407–421. 10.1111/j.1467-8519.2005.00452.x, PMID: 16222856

[ref46] Maynard Smith J. Price G. R. (1973). The logic of animal conflict. Nature 246, 15–18. 10.1038/246015a0

[ref47] Miller G. (2020). Social distancing prevents infections, but it can have unintended consequences. *Science Magazine*. Available at: https://www.sciencemag.org/news/2020/03/we-are-social-species-how-will-social-distancing-affect-us (Accessed February 10, 2021).

[ref48] Murphy K. Williamson H. Sargeant E. McCarthy M. (2020). Why people comply with COVID-19 social distancing restrictions: self-interest or duty? Aust. N. Z. J. Criminol. 53, 477–496. 10.1177/0004865820954484

[ref49] Naso R. C. (2020). Covid-19 and the free-rider problem. J. Psychol. Clin. Psychiatry 11:72. 10.15406/jpcpy.2020.11.00674

[ref50] Olson M. Jr. (1965). The logic of collective action: Public goods and the theory of groups. Cambridge, MA: Harvard University Press.

[ref51] Ostrom E. (2000). Collective action and the evolution of social norms. J. Econ. Perspect. 14, 137–158. 10.1257/jep.14.3.137

[ref52] Paakkari L. Okan O. (2020). COVID-19: health literacy is an underestimated problem. Lancet Public Health 5, e249–e250. 10.1016/S2468-2667(20)30086-4, PMID: 32302535 PMC7156243

[ref53] Pan Y. Fang Y. Xin M. Dong W. Zhou L. Hou Q.. (2020). Self-reported compliance with personal preventive measures among Chinese factory workers at the beginning of work resumption following the covid-19 outbreak: cross-sectional survey study. J. Med. Internet Res. 22:e22457. 10.2196/22457, PMID: 32924947 PMC7527164

[ref54] Perc M. Jordan J. J. Rand D. G. Wang Z. Boccaletti S. Szolnoki A. (2017). Statistical physics of human cooperation. Phys. Rep. 687, 1–51. 10.1016/j.physrep.2017.05.004

[ref55] Piraveenan M. Sawleshwarkar S. Walsh M. Zablotska I. Bhattacharyya S. Farooqui H. H.. (2020). Optimal governance and implementation of vaccination programs to contain the COVID-19 pandemic. ArXiv [Preprint]. Available at: http://arxiv.org/abs/2011.06455 (Accessed February 19, 2021). 10.1098/rsos.210429 PMC8188005 34113457

[ref56] Porterfield C. (2020). No-mask attacks: nationwide, employees face violence for enforcing mask mandates. *Forbes*. Available at: https://www.forbes.com/sites/carlieporterfield/2020/08/15/no-mask-attacks-nationwide-employees-face-violence-for-enforcing-mask-mandates/ (Accessed January 9, 2021).

[ref57] Prichard E. C. Christman S. D. (2020). Authoritarianism, conspiracy beliefs, gender and COVID-19: links between individual differences and concern about COVID-19, mask wearing behaviors, and the tendency to blame China for the virus. Front. Psychol. 11:597671. 10.3389/fpsyg.2020.597671, PMID: 33329265 PMC7732680

[ref58] Raihani N. J. Bshary R. (2015). The reputation of punishers. Trends Ecol. Evol. 30, 98–103. 10.1016/j.tree.2014.12.003, PMID: 25577128

[ref59] Ronay R. von Hippel W. (2010). The presence of an attractive woman elevates testosterone and physical risk taking in young men. Soc. Psychol. Personal. Sci. 1, 57–64. 10.1177/1948550609352807

[ref23] Ruan Z. Tang M. Liu Z. (2012). Epidemic spreading with information-driven vaccination. Phys. Rev. E Stat. Nonlin. Soft Matter Phys. 86:036117. 10.1103/PhysRevE.86.036117 23030990

[ref60] Sharma A. Menon S. N. Sasidevan V. Sinha S. (2019). Epidemic prevalence information on social networks can mediate emergent collective outcomes in voluntary vaccine schemes. PLoS Comput. Biol. 15:e1006977. 10.1371/journal.pcbi.1006977, PMID: 31120877 PMC6532839

[ref61] Simonov A. Sacher S. K. Dubé J.-P. H. Biswas S. (2020). The persuasive effect of Fox news: non-compliance with social distancing during the covid-19 pandemic. NBER. 10.2139/ssrn.3604214

[ref62] Sridhar D. Gurdasani D. (2021). Herd immunity by infection is not an option. Science 371, 230–231. 10.1126/science.abf7921, PMID: 33446540

[ref63] Tan L. K. Li N. P. (2020). The major factor behind Asia’s more effective handling of diseases. *The Business Times*. Available at: https://www.businesstimes.com.sg/opinion/the-major-factor-behind-asias-more-effective-handling-of-diseases/ (Accessed December 28, 2020).

[ref65] Tooby J. Cosmides L. Price M. E. (2006). Cognitive adaptations for n-person exchange: the evolutionary roots of organizational behavior. Manag. Decis. Econ. 27, 103–129. 10.1002/mde.1287, PMID: 23814325 PMC3693395

[ref66] Traulsen A. Röhl T. Milinski M. (2012). An economic experiment reveals that humans prefer pool punishment to maintain the commons. Proc. R. Soc. B Biol. Sci. 279, 3716–3721. 10.1098/rspb.2012.0937, PMID: 22764167 PMC3415907

[ref67] van Oosterhout C. Hall N. Ly H. Tyler K. M. (2021). COVID-19 evolution during the pandemic: implications of new SARS-CoV-2 variants on disease control and public health policies. Virulence 12, 507–508. 10.1080/21505594.2021.1877066, PMID: 33494661 PMC7849743

[ref68] van Prooijen J. -W. van Vugt M. (2018). Conspiracy theories: evolved functions and psychological mechanisms. Perspect. Psychol. Sci. 13, 770–788. 10.1177/1745691618774270, PMID: 30231213 PMC6238178

[ref69] Vardavas R. Breban R. Blower S. (2007). Can influenza epidemics be prevented by voluntary vaccination? PLoS Comput. Biol. 3:30085. 10.1371/journal.pcbi.0030085, PMID: 17480117 PMC1864996

[ref70] Weitz J. S. Beckett S. J. Coenen A. R. Demory D. Dominguez-Mirazo M. Dushoff J.. (2020). Modeling shield immunity to reduce COVID-19 epidemic spread. Nat. Med. 26, 849–854. 10.1038/s41591-020-0895-3, PMID: 32382154 PMC8272982

[ref71] Wilson S. (2020). Pandemic leadership: lessons from New Zealand’s approach to COVID-19. Leadership 16, 279–293. 10.1177/1742715020929151

[ref72] Wong J. Wong N. (2021). The economics and accounting for COVID-19 wage subsidy and other government grants. Pac. Account. Rev. 10.1108/PAR-10-2020-0189

[ref73] Yong J. C. Li N. P. Kanazawa S. (2020). Not so much rational but rationalizing: humans evolved as coherence-seeking, fiction-making animals. Am. Psychol. 10.1037/amp0000674 [Epub ahead of print] 33151700

[ref74] Yong J. C. Park G. Spitzmuller M. (2021). From the savannah to the corporate office: the evolution of teams. Small Group Res. 52, 33–67. 10.1177/1046496420960516

